# Do *perceived* working conditions and patient safety culture correlate with *objective* workload and patient outcomes: A cross-sectional explorative study from a German university hospital

**DOI:** 10.1371/journal.pone.0209487

**Published:** 2019-01-04

**Authors:** Heidrun Sturm, Monika A. Rieger, Peter Martus, Esther Ueding, Anke Wagner, Martin Holderried, Jens Maschmann, on behalf of the WorkSafeMed Consortium

**Affiliations:** 1 Institute of Occupational and Social Medicine and Health Services Research, University Hospital of Tübingen, Wilhelmstraße, Tübingen, Germany; 2 Institute of General Practice and Interprofessional Care, University Hospital of Tübingen, Osianderstr, Tübingen, Germany; 3 Institute for Clinical Epidemiology and Applied Biometry, University Hospital of Tübingen, Silcherstraße, Tübingen, Germany; 4 Department of Quality Management, Medical and Business Development, University Hospital of Tübingen, Hoppe-Seyler-Str, Tübingen, Germany; 5 University Hospital Jena, Medical Director, Bachstrasse, Jena, Germany; Nord University, NORWAY

## Abstract

**Background:**

Workload and demands on hospital staff have been growing over recent years. To ensure patient and occupational safety, hospitals increasingly survey staff about perceived working conditions and safety culture. At the same time, routine data are used to manage resources and performance. This study aims to understand the relation between survey-derived measures of how staff perceive their work-related stress and strain and patient safety on the one hand, and routine data measures of workload and quality of care (patient safety) on the other.

**Methods:**

We administered a written questionnaire to all physicians and nurses in the inpatient units at a German university hospital. The questionnaire was an amalgam of the Copenhagen Psychosocial Questionnaire (COPSOQ), the Copenhagen Burnout Inventory (CBI) scale to assess patient-related burnout of and portions of the Hospital Survey on Patient Safety Culture (HSPSC). Indicators from administrative data used to assess workload and patient-related work-strain were: amount of overtime worked, work intensity recording of nurses, cost weight, occupancy rate and DRG-related length of stay. Quality of care was assessed using readmission rates and disease-related length of stay. Univariate associations were tested with Pearson correlations.

**Results:**

Response rate were 37% (224) for physicians and 39% (351) for nurses. Physicians’ overtime correlated strongly with perceived **quantitative demands** (.706, 95% CI: 0.634 to 0.766), **emotional demands** (.765; 95% CI: 0.705 to 0.814), and perceived **role conflicts** (.655, 95% CI: 0.573 to 0.724). Nurses’ work-intensity measures were associated with decreasing physician job satisfaction and with less favorable perceptions of the appropriateness of staffing (-.527, 95% CI:-0.856 to 0.107). Both professional groups showed medium to strong associations between the morbidity measure (cost weight) and role conflicts; between occupancy rates and role clarity (-.482, 95% CI: -0.782 to -0.02) and predictability of work (-.62, 95% CI: -0.848 to -0.199); and between length of stay and internal team functioning (-.555, 95% CI: -0.818 to -0.101). Higher readmission rates were associated with lower perceived patient safety (-.476, 95% CI: -0.779 to 0.006), inadequate staffing (-.702, 95% CI: -0.884 to -0.334), and worse team functioning (-.520, 95% CI: -0.801 to -0.052). Shorter disease-related length of stay was associated with better teamwork within units (-.555, 95% CI: -0.818 to -0.101) and a lower risk of physician burnout (-.588, 95% CI: -0.846 to -0.108).

**Conclusion:**

Perceptions of hospital personnel regarding sub-optimal workplace safety and teamwork issues correlated with worse patient outcome measures. Furthermore, *objective* measures of overtime work as well as *objective* measures of workload correlated clearly with s*ubjective* work-related stress and strain. This suggests that objective workload measures (such as overtime worked) could be used to indirectly monitor job-related psychosocial strain on employees and, thus, improve not only staff wellbeing but also patient outcomes. On the other hand, listening to their personnel could help hospitals to improve patient (and employee) safety.

## Introduction

The working environment in German hospitals has seen significant changes over the last few years. Due to the introduction of a DRG-based reimbursement-system, economics have increasingly influenced organization of care. Between 2004 and 2016, whilst the number of hospital beds decreased from 531.3 thousand to 498.7 thousand, the number of inpatients increased from 16.8 million to 19.5 million, and the average length of stay diminished from 8.7 to 7.3 days. Nurse-to-patient ratios have decreased over time and care, currently to 17.9 per 1000 cases [1, 2]. Despite this, workloads have continuously increased.

Demands on hospital staff are growing for various other reasons too, such as increasing complexity of diseases due to demographic change [3–5], growing technical demands, and the need for new skills due to innovation [6]. Workload and work environment not only impact patient safety and quality of care [7], but also work-related safety of employees. Both, work-related strain and burnout rates of nurses in Germany have markedly increased. Burnout-rates doubled from about 15% in 1999 to 30.1% in 2011 [8, 9].

To guarantee patient and occupational safety in this challenging environment, appropriate psychosocial working conditions and strong leadership by hospital management and supervisors play central roles in counterbalancing increasing work demands [10–13]. The evidence for this relationship was reinforced by numerous studies using perceived working conditions in hospitals [14–20].

Managers increasingly utilise questionnaire-studies or regular staff surveys on *perceived* work-related stress and strain. Evidence on the importance of safety culture on patient outcomes in hospitals is growing as well [21, 22]. In the USA, the Agency for Healthcare Research and Quality has promoted regular monitoring of hospital safety culture since 2004 [23, 24]. To date, in Germany, regular safety-culture surveys and their use as a quality management tool have been less frequently applied [21].

Questionnaires looking at perceived working conditions offer more insights in organizational and managerial deficits and strengths than do crude administrative data: e.g. demands as defined in the demand-control-model by Karasek and Theorell, which include—in addition to the crude amount of work—the perceived pressure [25, 26]. Siegrist included responsibility, time-pressure, inconsistent demands on the “effort” side and recognition, job safety etc. on the “reward” side of his model [27]. Thus, job strain is always also dependent on, and can be counterbalanced by, the working environment. This in turn can be measured by established questionnaires [20, 28, 29].

Objective workload measures are based on administrative data such as nurse-patient ratio, occupancy levels [30] and turnover rates in terms of length of stay [31, 32]. The relationship between workload (in terms of appropriate staffing) and patient outcomes is well established [33–35], and evidence from Germany is growing [36, 37]. However, the political discussion concerning minimal levels of hospital personnel to ensure quality of care is ongoing, as the evidence seems still inconclusive.[38]

Administrative data might be more readily and regularly available but methodological shortcomings, such as completeness and validity, have been highlighted [39–41]. Also, its relation to staff-reported work-strain is still unclear [41].

Therefore, understanding the association between the two measures (perceived and objective) might enable better prioritization of improvements to working conditions and patient (and occupational) safety. However, there is little evidence for the correlation between *objective* workload measures and *perceived* stress and strain. [41]

We aim to assess the relation between perceived stress and strain of physicians and nurses and their workload, as measured by routine data. In addition, we want to test if perceived patient safety corresponds with objective non-disease-specific patient-safety and quality-of-care measures available in routine data. By doing so, we add to the limited existing literature concerning performance quality has rarely been linked to staff perceptions [42, 43]. Correlations between workload and patient outcomes or patient safety have mostly been assessed for specific diseases like hip fracture and myocardial infarction [44, 45].

## Methods

### Design

This study is part of a cross-sectional, multicenter, mixed-methods study (*working conditions*, *safety culture and patient safety in hospitals–what predicts the safety of the medication process* WorkSafeMed) performed within two German university medical centers. The aim is to investigate the relationship between working conditions, safety culture and patient safety [20, 46]. Perceived job stress and strain was assessed by means of a questionnaire study.

In an explorative approach the sub-project presented in this paper connected questionnaire results with routine data in order to explore the correlations between those two data collection methods. We therefore related questionnaire scales measuring *workload*, *safety culture* and *perceived safety* with available administrative data assessing *workload* and *quality-of-care*.

### Questionnaire study

#### Questionnaire

In order to assess the psychosocial working conditions for hospital staff and the prevalent safety culture, we developed a questionnaire based on validated and commonly used instruments (Copenhagen Psychosocial Questionnaire (COPSOQ)) [28, 47], one adapted scale to assess client-related burnout of the Copenhagen Burnout Inventory (CBI) [48] and the German version of the Hospital Survey on Patient Safety Culture (HSPSC-D) e.g. [23]). Overall, the questionnaire consisted of 36 scales with 153 items, of which 25 scales were analyzed in this study (Table 1). Mostly items were rated on a 5-point Likert-scale of either agreement or frequency. Prior to data collection, the final survey underwent a pre-test with 4 physicians and 8 nurses using cognitive think aloud interviews. Depending on the wording of items within each scale, maximum values can be positive (high = positive) or negative (high = negative). For example: a high value for “influence at work” is positive while a high value for “quantitative demands” is negative. Questionnaire data was imputed: when respondents had >30% missings per scale, then data were imputed with NORM 2.03 software using the Expectation-Maximization-algorithm [12, 49]. Organizational administrative data was not imputed, as only data from available organizational units was used. (Details are described elsewhere [12, 20, 50]).

**Table 1:** Overview of scales and items of the questionnaire.

Topic	Instrument	Scales / indices / single items	Interpretation
Psychosocial working conditions	COPSOQ^1^ [28, 47]	Quantitative demands (scale, 4 items)	High = negative
		Emotional demands (scale, 3 items)	High = negative
		Work-privacy-conflict (scale, 5 items)	High = negative
		Influence at work (scale, 4 items)	High = positive
		Degree of freedom at work (scale, 4 items)	High = positive
		Job satisfaction (scale, 7 items)	High = positive
		Predictability (scale, 2 items)	High = positive
		Role clarity (scale, 4 items)	High = positive
		Role conflicts (scale, 4 items)	High = negative
		Social support (scale, 4 items)	High = positive
		Social relations (scale, 2 items)	High = positive
		Sense of community (scale, 3 items)	High = positive
		Meaning of work (scale, 3 items)	High = positive
		Workplace commitment (scale, 4 items)	High = positive
		Quality of leadership (scale, 4 items)	High = positive
	TLI short^3^ [51]	Transformational leadership (scale, 6 items)	High = positive
	adapted from CBI^2^ [48]	Patient-related burnout (scale, 6 items)	High = negative
Patient safety dimensions	HSPSC-D^3^[46]	Teamwork within units (scale, 4 items)	High = positive
		Teamwork across units (scale, 4 items)	High = positive
		Handoffs and transitions (scale, 4 items)	High = positive
		Frequency of event reported (scale, 3 items)	High = positive
		Overall perceptions of patient safety (scale, 4 items)	High = positive
		Patient safety grade (single item)	Low = positive
		Staffing (scale, 4 items)	High = positive

^1^COPSOQ scales: range1-4 or 1–5

^2^CBI scale: range 1–5. Before calculating scale scores of COPSOQ and CBI (Copenhagen Burnout Inventory), scales were transformed into scores ranging from 0 (minimum value, “do not agree at all”) to 100 points (maximum value, “fully agree”).

^3^TLI short scale and HSPSC-D (Hospital Survey on Patient Safety Culture): range 1–5.

#### Scales for work-related stress and strain

From COPSOQ, HSPSC-D and CBI (n = 17 scales): quantitative demands, emotional demands, influence at work, degree of freedom at work, meaning of work, workplace commitment, predictability, role clarity, role conflicts, social support, social relations, sense of community, job satisfaction, quality of leadership, work-privacy-conflict, staffing, adapted scale to assess patient-related burnout.

#### Scales for patient safety culture and perceived safety

(HSPSC-D) (n = 8 scales): teamwork within units, teamwork across units, staffing, handoffs & transitions, safety grade in the medication process, overall perceptions of patient safety, patient safety grade, frequency of events reported. (For an overview of all used scales see Table 1)

#### Sample

Between March and June 2015, all physicians and nurses (including nursing aids and nurses in training) from units with more than 500 inpatients per year (excluding units with low medication use, special settings or not enough inpatients: intensive care, psychiatry, ophthalmic clinic, radiology units, dentistry and oral and maxillofacial medicine) were actively invited to take part in the survey (n = 1,502) (see also Wagner et.al. [20]). In this analyses we were able to include questionnaires returned from 224/607 physicians (37%) and 351/ 895 nurses (39%). They worked in 40 units distributed among 23 departments within a German university hospital (Table 2). Results from the survey were available for physicians on department level and for nurses at unit-level.

**Table 2:** Questionnaire study–response rates of participating staff.

	Questionnaires distributed (n)	Questionnaires analyzed (n)	Response rates (%)	Age years (min/max)	Gender (% male)
Total (20 departments/40 units)	1,502	575	38.3%		
Physicians	607	224	36.9%	36.8 (25/63)	50.5
Nurses	895	351	39.2%	41.1 (24/61)	16.8

### Administrative data

The following indicators were used to assess **workload**: To assess physicians’ workload we used *worked overtime*, available on department-level (n = 18). For workload of nurses we used *work intensity*, an administrative measure (Leistungserfassung Pflege, *LEP)* established in Germany, Switzerland, Austria and Italy, that monitors the proportion of non-patient-related activities in relation to available staff on a daily bases [52, 53]. It is assumed that more direct patient-related working time correlates with higher workload [54, 55]. This parameter was available for 30 units / 11 departments (Table 3).

**Table 3:** Number of units or wards analyzed in correlations.

Available Data	*Morbidity* *(cost-weight)*	*Occupancy rates*	*Disease related length of stay (LOS)*	*Readmission rates*	*Work-intensity (nurses)*	*Over-hours (physicians)*	*Available questionnaire data*
**Nurses (units)**	38	38	38	38	30	16	40
**Physicians (departments)**	15	15	15	15	11	15	20
Professions combined (departments*)	17	17	17	17	-	-	20

Units for comparison of nurses, departments for comparison of physicians

*Averaged data between physicians and nurses was used in correlations. For some departments only nursing data was available, which was used for the computation of “Professions combined”.

For **patient-related work strain** routinely available and commonly used proxies are used: length of stay, occupancy rates and morbidity [33]. For the latter *“relative cost weight”* was used, which takes length of stay and resource utilization related to the individual diagnosis related group (DRG) into account and reflects the adjusted DRG-based diagnosis. *DRG-related length of stay (LOS)*, mainly collected for assessing quality of care, is also known to correlate with severity of disease, therefore we used it in this explorative approach as an additional proxy for work strain [56]. In addition, *occupancy rates* [30] were assumed to reflect work strain, with shorter length of stay indicating higher work-intensity [33].

The following indicators were used to assess quality:

As an established measure indicating complications, we used *average 30-day readmission rates for all causes* [57–59]. In addition, we used *DRG-related length of stay (LOS)* as a proxy for quality of care, as longer than average LOS may reflect complications and thus potential quality problems [31, 36].

Data availability: Routine data were collected during the first 6 months of 2015 from all clinical departments, where both data sources (questionnaire and routine data) were available. Data was collected either pseudonymously (patient related data) or aggregated (hospital performance data). Analysis was performed on an aggregate level, using averaged data per organizational unit (Table 3). Patient-level administrative data was available from all units and average per unit was used for analysis. Therefore, the sample size differs between individual analyses (Table 3).

### Statistical analysis

Data was analyzed as averages per organizational unit. All variables were examined for normal distribution. In cases of right skewed distributions logarithmic transformation led to an acceptable fit to the normal distribution. Univariate association between questionnaire results and routine data were tested with Pearson correlations. Correlations were tested separately for nurses and physicians, as well as for both professional groups together.

First, we built defined three groups of theoretical associations (1. perceived work-related stress and strain and clinical data related to workload; 2. perceived work-related stress and strain and patient-related workload; 3. Perceived patient safety and administratively measured quality of care). Next to 11 predefined study questions relating individual questionnaire scales to individual routine data measures (see Tables 2–5, results in bold), in an explorative approach, we tested all correlations between subjective and objective work strain data as well as between subjective and objective quality and safety data.

**Table 4:** Correlations between clinical data related to workload and perceived work-related stress and strain.

			*Workload measures (processes)*			
*Scales*^*+*^	*Orientation of scales*	*Professional group*	*Work Intensity* *(nurses)*		*Worked Overtime (physicians)*	
			*Corr*.*Coeff*. *(CI)*	*p*	*Corr*.*Coeff*. *(CI)*	*p*
**staffing (HSPSC-D)**	High = positive	Physicians	-.527 (-0.856 to 0.107)	0.095		
**job satisfaction**	High = positive	Physicians	-.582 (-0.876 to 0.027)	0.060		
**quantitative demands**	High = negative	Physicians			.706 (0.303 to 0.895)	0.003
**work privacy conflict**	High = negative	Physicians			.642 (0.193 to 0.869)	0.010
*emotional demands*	*High = negative*	*Physicians*	* *		.*765 (0*.*416 to 0*.*918)*	*0*.*001*
*role conflicts*	*High = negative*	*Physicians*	* *		.*655 (0*.*215 to 0*.*874)*	*0*.*008*
*influence at work*	*High = positive*	*Nurses*	.*336 (-0*.*028 to 0*.*621)*	*0*.*070*	* *	

All correlations tested under the primary hypothesis as well as explorative correlations with p< 0.1 are reported; Pearson Corr. Coeff of < .30 is considered a small (irrelevant) effect, .30 to .50 medium (medium relevance) and .50 to 1 strong effect (high relevance). For Bonferroni-correction p needs to be multiplied by 11. Purely explorative correlations scales are presented in italics, primary study questions in bold.

+ Scales are based on COPSOQ, if not mentioned otherwise.

**Table 5:** Correlations between perceived work-related stress and strain and patient-related workload.

			*Patient-related workload*			
*Scales*^*+*^	*Orientation of scales*	*Professional group*	*cost weight* *(Morbidity)*		*Occupancy rates*	
			*Corr*.*Coeff*. *(CI)*	*p*	*Corr*.*Coeff*. *(CI)*	*p*
**quantitative demands**	**High = negative**	**Physicians**	**.446 (-0.86 to 0.78)**	**0.095**	** **	
*role conflicts*	*high = negative*	*combined*	.*471 (-0*.*12 to 0*.*776)*	*0*.*056*	* *	
*role clarity*	*High = positive*	*Physicians*	.*449 (-0*.*82 to 0*.*782)*	*0*.*093*	* *	
*Patient related Burnout (adapted from CBI)*	*High = negative*	*Physicians*	.*48 (-0*.*43 to 0*.*796)*	*0*.*070*	*-*.*496 (-0*.*804 to* .*022)*	*0*.*060*
*Team within units (HSPSC-D)*	*High = positive*	*combined*	*-*.*436 (-0*.*758 to 0*.*56)*	*0*.*080*	* *	
*Social support*	*High = positive*	*Nurses*	.*284 (-0*.*39 to 0*.*553)*	*0*.*084*	* *	
*role clarity*	*High = positive*	*combined*	* *		*-*.*482 (-0*.*782 to -0*.*02)*	*0*.*050*
**job satisfaction**	**High = positive**	**Physicians**	** **		**.48 (-0.043 to 0.796)**	**0.070**
**job satisfaction**	**High = positive**	**Nurses**	** **		**-.277 (-0.548 to 0.047)**	**0.092**
**Staffing (HSPSC-D)**	**High = positive**	**Nurses**	** **		**-.272 (-0.544 to 0.052)**	**0.098**
*Predictability*	*High = positive*	*Nurses*	* *		*-*.*282 (-0*.*552 to 0*.*041)*	*0*.*086*
*Predictability*	*High = positive*	*combined*	* *		*-*.*62 (- =* .*848 to -0*.*199)*	*0*.*008*
**work privacy conflict**	**High = negative**		**n.s.**		**n.s.**	

All correlations tested under the primary study question **as well as explorative correlations with** p< 0.1 are reported; Pearson Corr. Coeff of < .30 is **considered** a small (irrelevant) effect. .30 to .50 medium (medium relevance) and .50 to 1 strong effect (high relevance). For Bonferroni-correction p needs to be multiplied by 11. Purely explorative correlations scales are presented in *italics*, primary study questions in **bold.** Cost weight: DRG-related morbidity score of a unit. Higher cost-weight indicates higher morbidity. Low occupancy rates indicate lower workload.

^***+***^Scales are based on COPSOQ, if not mentioned otherwise.

With this aim, data were aggregated on unit level for correlations between patient-related and nurse-related data, and on department level for patient-related and physician-related data. The level of significance was set at 5% (two-sided), trends were reported at a 10% level. Without correction of multiple testing, correlations of 0.67 (n = 15) / 0.64 (n = 17) and 0,45 (n = 38) could be identified (i.e. significance for null hypothesis “correlation = 0”) with 80% power. Using Bonferroni correction for the 11 predefined study questions (Table 2: four; Table 3: four; Table 4: two and Table 5: one primary study question), correlations of 0.79 (n = 15) / 0.76 (n = 17) and 0,56 (n = 38) could be identified, when correction with factor 11 was applied.

All correlations tested for the primary study questions are reported. For explorative correlations only effects with a significance level of 10% or lower are reported.

In interpreting the coefficient in terms of effect size, we followed the recommendations of Bühner and Ziegler [60], where < .30 is a small (irrelevant) effect, .30 to .50 is a medium effect (medium relevance), and .50 to 1 is a strong effect (high relevance).

All statistical analysis was performed using IBM Statistics SPSS (Version 23) [61] for Windows.

### Ethics and confidentially issues

Ethics approval was obtained from the *Independent Ethics Committee (IEC) of the University of Tübingen* (697/2014VF, 12-2-2014). (Written) informed consent was sought from questionnaire participants, who were informed that the study was voluntary and that they could withdraw at any time. The questionnaire was distributed in every organizational unit accompanied by an explanation of privacy, data handling and voluntary participation. These explanations were also attached in front of the questionnaire. By sending back the questionnaire, the employee demonstrated his willingness to participate. This was consented by the employee committee. The Ethics committee waived the need for further consent, as the data was collected anonymously (697/2014VF, 12-2-2014). Questionnaire data was analyzed anonymously. Routine controlling data for hospital performance was collected on an aggregated level and was therefore anonymous. Routine data on patients was available on an individual level (e.g. length of stay, cost weight), however accessible only as pseudonymized data. For analysis, aggregated data on organizational unit was used. As these analyses were considered part of quality assurance (based on the regional legislation (LDSG; LKHG §45) by the data-security officer, no additional individual consent of patients was sought. (Writing from 4-16-2015/SR).

## Results

The questionnaire was completed by 224 (37%) physicians and 351 (39%) invited nurses (Table 2). The average age of physicians was 37 years; about 16% were specialists, and 18% were interns. The mean age of nurses was 40 years; 84% were female, and nearly 62% were certified.

We were able to include patient related routine data from overall 11,095 cases in 23 departments. Based on data availability, correlations could be calculated for nurses from 38 units, for physicians from 15 departments. Included cases per correlation may vary slightly according to available data. For two departments, only nursing (survey) data was available which was then used for the average values used in the correlation for both professions (Table 3).

### Correlations of perceived stress and strain and objective workload

The strongest (significant) correlations were found for **physicians’ overtime hours** and their perceived *quantitative demands (*.706, p = 0.003); *emotional demands* (.765, p = 0.001); and physicians’ perceived *role conflicts* (.655, p = 0.008). In addition, we found that increasing levels of doctors’ overtime correlated with increasing *work-privacy conflicts* (.642; p = .010).

The objective workload of the nursing staff (**work intensity**) did not show statistically significant correlations with any of the questionnaire scales reflecting quantitative or emotional demands. There was, however, a tendency for nursing workload to correspond with higher subjective *influence at work* (.336, p = 0.07).

On the other hand, increasing nursing workload was negatively correlated with physicians’ *job satisfaction* (-.582, p = 0.060) and their perceptions of appropriateness of *staffing* (-.527, p = 0.095) (Table 4).

### Correlations of work strain related to patient morbidity

We hypothesized, that sicker patients (as reflected by cost weight) induce higher work strain and workload for physicians and nurses. For **cost weight (morbidity)** (Table 5) we could only detect statistically non-significant trends relating to perceived work-related stress or strain except for regarding *role conflicts* (.471; p = 0.056). In line with that, both professional groups also showed weak inverse correlations between the morbidity measure and *teamwork within units*, which indicates how well team functioning is perceived. On the other hand, physicians’ answers indicated increasing *role clarity* with increasing morbidity of patients.

Physicians’ answers concerning *patient-related burnout (CBI)* showed a tendency for burnout to increase with patient morbidity (.48; p = 0.070). Physicians also indicated subjectively higher *quantitative demands* when caring for sicker patients (.446; p = 0.095), while nurses did not.

In contrast, nursing staff showed a slightly positive correlation between *social support* and the morbidity measure (.284; p = 0.084) (Table 5).

**Fig 1:**
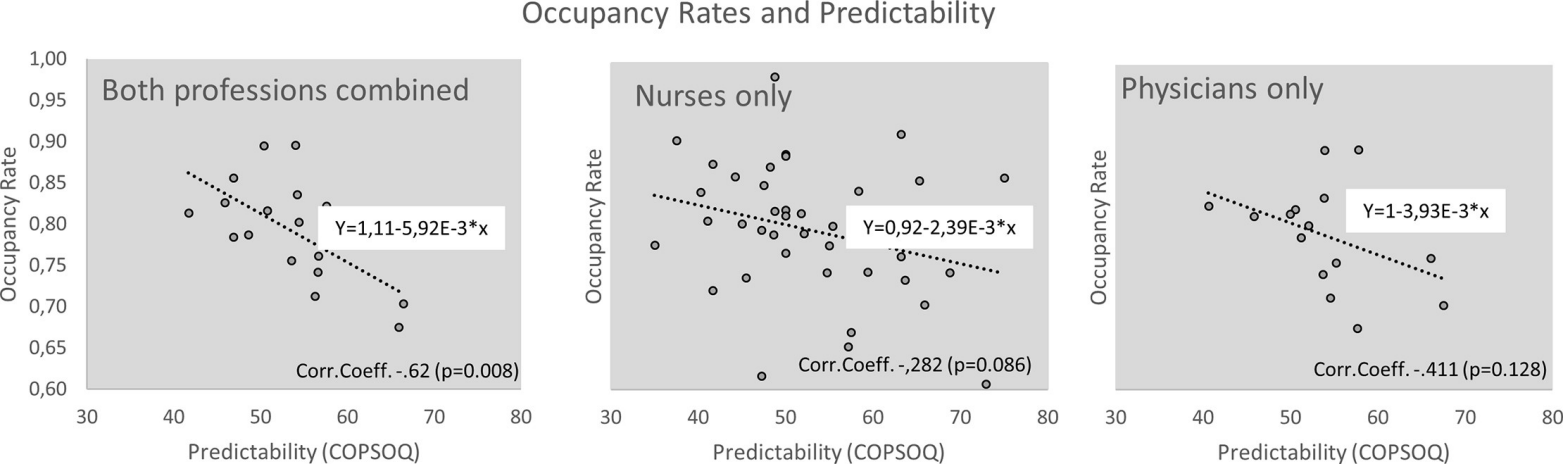
Correlations: Predictability vs. occupancy rates for different professional groups.

Statistically significant inverse correlations between **occupancy rates** and perceived psychosocial stress and strain were found for *role clarity* (-.482; p = 0.050) and *predictability* (-.62; p = 0.008) in both professional groups (Table 5 and Fig 1). The scale *role clarity* refers to how well defined the professional tasks and roles are perceived.

Opposing trends between the professional groups could be detected for *job satisfaction* when occupancy rates were higher: while nurses`job satisfaction decreased (0.48; p = 0.070), physicians’ job satisfaction increased (-.277; p = 0.092). While physicians’ *job satisfaction* increased in a fully occupied unit environment, physicians’ *burnout*-scale decreased (-.496; p = 0.060) with higher occupancy rates.

### Correlations between perceived patient safety and quality of care

We also tested the correlation between quality of care reflected in routine data and perceived patient safety and related scales (Table 6).

**Table 6:** Quality of care and perceived patient safety.

*Scales*	*Orientation of scales*	*Professional group*	*Quality of Care*	
			*Readmission rates*	
			*Corr*.*Coeff*. *(CI)*	*P*
**patient safety grade (single item) (HSPSC-D)**	**High = positive**	**combined**	**-.476 (-0.779 to 0.006)**	**0.053**
**overall perception of patient safety (HSPSC-D)**	**High = positive**	**combined**	**-.443 (-0.762 to 0.048)**	**0.075**
*frequency of events reported (HSPSC-D)*	*High = positive*	*Physicians*	*-*.*590 (-0*.*846 to -0*.*111)*	*0*.*021*
*safety grade in the medication process (single item) (HSPSC-D)*	*High = positive*	*Nurses*	*-*.*335 (-0*.*591 to -0*.*017)*	*0*.*040*

All correlations tested under the primary study question as well as explorative correlations with p< 0.1 are reported; Pearson Corr. Coeff of < .30 is considered a small (irrelevant) effect, .30 to .50 medium (medium relevance) and .50 to 1 strong effect (high relevance). For Bonferroni-correction p needs to be multiplied by 11. Purely explorative correlations scales are presented in italics, primary study questions in bold. Readmission rate: average 30-day readmission rates for all causes. Higher readmission rates can indicate quality problems.

While only nurses’ judgments of *medication safety* (-.335; p = 0.040) correlated significantly with the frequency of **readmissions**, perceived *patient safety grade* (-.476; p = 0.053) correlated with readmissions for all staff. The same trend is visible for the multi-item scale for *overall perception of patient safety* (-.443; p = 0.075).

Physicians’ answers to *frequency of events reported* correlated negatively with readmissions.

**Length of stay** did not show any correlation with staff perceptions of patient-safety culture.

The strongest (negative) correlation was found between **readmission** rates and *staffing* by both professional groups (-.702; p = 0.002): with higher readmission rates, the personnel situation was increasingly perceived as insufficient (Table 7)

**Table 7:** Quality of care and work-related strain.

			*Quality of Care*			
*Scales*	*Orientation of scales*	*Professional group*	*Readmission rates*		*Length of Stay*	
			*Corr*.*Coeff*. *(CI)*	*P*	*Corr*.*Coeff*. *(CI)*	*p*
hands-off and Transition (HSPSC-D)			n.s.		n.s.	
*teamwork within units (HSPSC-D)*	*High = positive*	*Physicians*	*-*.*449 (-0*.*782 to 0*.*082)*	*0*.*093*	* *	
*teamwork within units (HSPSC-D)*	*High = positive*	*combined*	* *		*-*.*555 (-0*.*818 to -0*.*101)*	*0*.*021*
*teamwork across units (HSPSC-D)*	*High = positive*	*combined*	*-*.*520 (-0*.*801 to -0*.*052)*	*0*.*032*	* *	
*staffing (HSPSC-D)*	*High = positive*	*combined*	*-*.*702 (-0*.*884 to -0*.*334)*	*0*.*002*	* *	
*patient-related burnout (adapted from CBI)*	*High = negative*	*Physicians*	* *		*-*.*588 (-0*.*846 to -0*.*108)*	*0*.*021*

All correlations tested under the primary study question as well as explorative correlations with p< 0.1 are reported; Pearson Corr. Coeff of < .30 is considered a small (irrelevant) effect, .30 to .50 medium (medium relevance) and .50 to 1 strong effect (high relevance). For Bonferroni-correction p needs to be multiplied by 11. Purely explorative correlations scales are presented in italics, primary study questions in bold Readmission rate: average 30-day readmission rates for all causes. Higher readmission rates can indicate quality problems. DRG-related length of stay (LOS): longer than average LOS may reflect complications.

Both professional groups also showed a negative correlation between readmissions and the perceived *teamwork across units* (-.520; p = 0.032). Physicians’ answers indicated the same for *teamwork within units* (-.449; p = 0.093).

For both professional groups, better *teamwork within their unit* was correlated with decreasing patients´ **length of stay** (-.555; p = 0.021). In addition, when patients´ stays were shorter than expected, physicians showed a higher risk of patient-related burnout (*CBI*) (-.588; p = 0.021)(Table 7).

## Discussion

The main research question was to explore as to whether perceived work stress and strain correlated with workload measures of routine data. Secondly, we wanted to understand as to whether perceived patient safety culture and perceived patient safety are correlated with routine data reflecting patient outcomes. Our results suggest that objectively measured workload is associated with staff members´ perceived work-related psychosocial stress and strain; secondly, the results showed that perceived worse elements of safety culture (namely teamwork) as well as patient-related burnout are associated with worse patient-related outcomes as measured by readmissions and longer stays. Also, perceived overall patient safety correlates with patient outcome measures.

This supports the hypothesis that an increased objective work load can lead to more job-related stress and strain, which in turn can affect not only staff wellbeing, but also patient outcomes. Thus, improving working conditions could directly improve patient outcomes. The findings underline the importance of strategies to focus on safety culture in hospitals. Objective workload itself could be monitored by using routine data.

### Workload–objective and perceived

Firstly, physicians’ workload as measured in documented **overtime** corresponded strongly with perceived quantitative demands. Obviously, the perception of the required pace and the inability to complete work (during usual working hours) as depicted by the scale workload, is paralleled by actual worked overtime. In addition, physicians’ worked overtime corresponded with *role conflicts* and *emotional demands*. Therefore: not being able to perform work the way it should be, perceived contradictory or unnecessary demands and emotionally stressful situations go along with more worked overtime by physicians. Organization and team functioning not only play a major role in perceived workload but problems stemming from disorganization and deficiencies in teamwork may trigger costly and potentially unnecessary overtime. The relationship between teamwork, staff well-being and quality of care is well established [62, 63]. Both, overtime and perceived work-related stress and strain have been shown to negatively impact wellbeing of employees. Jeffs and colleagues highlighted the crucial role of strong and caring leaders, who create a supportive work environment as a central factor to minimizing overtime and absenteeism [64] Ochsmann et.al. highlighted, that overtime work and lack of supervisors’ feedback predict strain and recovery in young doctors. Our findings supported this; we noted that worked overtime corresponded with more *work-privacy conflicts* which might be explained by the fact that overtime decreases time for family responsibilities and private life in general.

From a management perspective, increasing worked overtime in a given unit might therefore point to a suboptimal working environment, especially if objective workload does not tally. Our results support the notion that unclear or conflicting roles and tasks, a lack of clear standards, and inefficient team communication can constitute potential barriers to smooth work processes. As a result, staff may spend more time coping with their tasks. Therefore, simply **monitoring overtime** in relation to objective workload could alert management to potential organizational shortcomings and without the need to survey personnel. Managers might find they can reduce costs by offering support measures, such as supervision of staff, that alleviate emotional stress and support optimal team function [65].

In both professional groups increasing objective **workload** (measured with cost-weight and occupancy rates) showed associations with perceived poorer team functioning, increasing role conflicts, and lack of role clarity and predictability of work. Perceived deficits included less clearly described tasks and professional roles, reduced ability to predict job tasks and changes and more tasks that staff perceived as unnecessary (such as documentation). Thus, higher work-load and increasing pressure seem to make process-related problems more noticeable for staff or, in turn process-related problems are more likely to occur when workload is high. The importance of the team is also underlined by the fact that physicians showed lower *job satisfaction* and indicated insufficient *staffing*, when nurses’ work-intensity scores were higher. Thus, in our study we could show a relation between objective and perceived workload-measures. Kalisch and colleagues also found correlations between nurse-reported staffing adequacy and workload, and empirically derived working hours [41] but concluded, that the correlations strongly depend on the available measures.

On the other hand, higher **morbidity-related demands** correlated with better perceived *social support*, greater *influence at work* among nurses and increasing *role clarity* among physicians, indicating that higher demands require or induce better ways to cope, whether because of mutual support or because of clearer definitions of the scope of work.

Physicians’ answers of perceived *work-related strain* correlated to patient and process related objective workload measures (Table 4 and Table 5), while nurses’ perceived work-related strain was less linked to routine data: Work intensity, with the exception of perceived *influence at work*, did not correlate with any scale of perceived work-related stress and strain, which might be due to the measure itself. Only occupancy rates were linked to perceived adequate *staffing*, *predictability* and *job satisfaction*, which decreased with higher occupancy rates. Interestingly though, physicians appear to be more satisfied with higher patient throughput, which may link to a perceived improvement in efficiency [66]. This is also in line with the findings of our crude questionnaire results, where physicians seem to evaluate their working conditions more positively than nursing staff. Especially the scales *degree of freedom at work and possibilities for development*, *meaning of work* had been assessed better by physicians [see additional material]. These findings also mirrors the different job types of the demand-control model of Karasek and Theorell [26], where jobs with high demands and at the same time high freedom and “decision latitude” result in “active jobs” with high productivity. Physicians certainly fit better into this category than do nurses.

There is a difference between the professional groups; physicians could be more affected by the severity of illness and economical strains, while nurses are more affected by high patient density and high throughput. The pressure on physicians confronted with increasing morbidity might be especially high because they have dual responsibility for medical and economic outcomes. Sick patients and economic constraints put pressure on work-processes, which in turn can induce conflicts within a team. Underlying economic constraints are especially likely to require difficult and disputable decisions. There is growing evidence that such economic constraints influence medical decision-making, and at times may lead to conflicting requirements [67–70]. This can also explain the increasing patient-related burnout rates among physicians when patients’ stays are shorter than expected for their conditions.

The cycle is a self-perpetuating one as physicians’ performance dwindles with burnout [71]. Also, the RN4Cast study showed a constant correlation between work-environment and burnout-rates of nurses in all participating countries [8, 9]. The feeling of being unable to influence one’s own work situation increases stress levels (including burnout) and decreases efficiency, while freedom of work organisation and possibilities to participate in decision making promote human functioning [72]. Job dissatisfaction is associated with decreased staff productivity [73, 74], whereas readily available social support (“job resources”) fosters motivation and commitment [29, 75, 76]. Kieft defined a healthy work environment as “a work setting in which nurses are able to both achieve the goals of the organization and derive personal satisfaction from their work” [23, 77]. Management and leadership therefore play a pivotal role in providing healthy and supportive work environments. Quality of leadership has been found to relate to job performance [78], job satisfaction and retention of staff [79]. High quality leadership includes sound communication processes and participation in decision making of staff [19, 80, 81].

In summary: monitoring workload through routine-data (especially with respect to physicians) can help to detect stressors early and prevent loss of productivity and performance. Our results support the notion that management can improve efficiency and outcomes, whilst reducing overtime, by focussing on the team, The use of clear standards, defined roles and tasks is paramount and strong leadership with a focus on a healthy working environment is a prerequisite.

### Working conditions and quality of care

The significant association between (perceived) appropriateness of staffing and patient outcomes in our data is consistent with the robust evidence, that patient to nurse ratios influence hospital mortality rates (e.g. [7]). In a study using discharge data from patients undergoing common surgeries in 300 hospitals in nine European countries, Aiken calculated a 7% increase in 30-day hospital mortality when nurses' workload increased by one patient [82]. Recently this topic has been taken up by German health policymakers when discussing minimal staffing levels for nurses in hospitals [83].

However, investing in staffing might not be sufficient. We found the quality indicators (readmission rates as well as length of stay) to be moderated by teamwork as much as by staffing. This relationship is in line with the work of Mark et.al. who found that nurses’ perception of quality of care is determined (amongst others) by leadership, staffing, resources, effective communication/collaboration, environment/culture and simplicity [84]. On the other hand, shorter length of stay, an indicator of better quality of care, correlated with increased physicians’ patient-related burnout. This would suggest that the workload effects of *shorter length of stay* are stronger than its quality effects. There is evidence that better physician wellbeing improves both the physicians’ health and quality of care [85]. Wallace [74] suggested that hospitals should therefore routinely monitor physician wellness. In our results, better teamwork correlated significantly with increased quality with both quality of care measures (fewer readmissions and shorter lengths of stay) and for both professional groups. Thus, as has been shown in other studies [63, 86, 87] our findings support the notion that good teamwork and communication can contribute to better outcomes. The converse is also true; difficulties in communication and teamwork can lead to more complications. More reported events correlated with fewer readmissions, indicating better quality of care when physicians used critical events reporting more frequently. This relationship does not seem to be fully explained by changes in individual processes but most likely results from a more general change. This is supported by the findings from Anderson [88] in which staff perceived that the positive effect of incident reporting was also due to changing staff attitudes and knowledge.

### Perceived and measured patient safety

All our questionnaire scales related to perceived patient safety turned out to correlate as expected with higher readmission rates but not with length of stay. This may indicate that *length of stay* does not capture patient safety or quality of care as well as *readmission rates*, because it also reflects workload. At the same time, these results show that staff perceptions of patient safety seem to accurately reflect patient safety in their working environment.

### Limitations and strengths

This study was an exploratory approach, therefore hypothesis driven rather than confirmatory. We were therefore rather unselective in our correlations. For the same reason, no prior calculation of sample size was performed and results of correlation analyses are shown without corrections for multiple testing. Although we relied on analysis of available administrative parameters, the questionnaire data was derived with well-established instruments. In addition, whilst the study included a broad range of hospital units (i.e. medical and surgical specialties), the data is limited to a single German university hospital, thus representing a specific setting. This is particularly relevant because administrative variables, such as overtime, are dependent on local policy and the regulatory framework. This cross-sectional study only analyzed correlations and therefore causal relations cannot be assessed. However, the associations we noted are supported in large part by existing literature.

Further research is required to understand how routine data can be effectively used to inform management of prevailing structural team-related problems.

## Conclusions

We showed a link between (objective) workload measures and perceived stress and strain, which, in turn, is known to correlate with work and patient safety as well as safety culture. We highlighted that o*bjective* worked overtime as well as *objective* work-load measures correlated clearly with s*ubjective* work-related stress and strain, which suggests that monitoring workload in relation to overtime may be a useful tool to detect problems in team and leadership dynamics that may impede smooth work processes. This study has provided proof of concept. Our indicators fall short as proven final indicators, but we suggest pursuing the use of routine data for monitoring work-related stress and strain. Routine data, in contrast to questionnaire data, are readily available and therefore monitoring is easier to implement. To further test the responsiveness of the indicators, longitudinal assessments and different settings should be analysed.

In addition, patient outcomes were related to perceived patient safety as well as to perceived team performance. Taken together, these findings suggest that objective increased workload can lead to more job-related stress and strain, which in turn can impact not only staff wellbeing but also patient outcomes. Thus, improving working conditions could directly improve patient outcomes. This adds further weight to the importance of strategies that focus on safety culture in hospitals.

## Supporting information

S1 Dataset Data both professions (SAV). (SAV)

S2 Dataset Data physicians (SAV). (SAV)

S3 Dataset Data nurses (SAV). (SAV)

## Abbreviations

WorkSafeMed: *working conditions*, *safety culture and patient safety in hospitals–what predicts the safety of the medication process*
LOS: disease related length of stay
DRG: diagnose-related group
COPSOQ: Copenhagen Psychosocial Questionnaire
HSPSC-D: German version of the Hospital Survey on Patient Safety Culture
CBI: Copenhagen Burnout Inventory
